# The Book World of Medicine and Science

**Published:** 1902-10-04

**Authors:** 


### The Book World of Medicine and Science.

**The Practitioner's Handbook of Diseases of the Ear and Naso-pharynx.** By H. Macnaughton-Jones, M.D., M.Ch., F.R.C.S. I. & Ed.; W. H. R. Stewart, F.R.C.S.Ed.; William Milligan, M.D., C.M.; Herbert Tilley, M.D., B.S., F.R.C.S.Eng.; Ambrose Birmingham, M.D., F.R.C.S.I., F.R.U.I.; and Robert Dwyer Joyce, F.R.C.S.I., M.R.C.S. With 182 illustrations and seven plates. Sixth edition. (London: Baillière, Tindall, and Cox. 1902. Price 10s. 6d. net.

With such a galaxy of talent on the title-page we expect to find this a good and useful book and we have not been disappointed. That specialists will find new light in its pages we do not imagine; but there can be no doubt that the practitioner who wishes, not only to obtain a general survey of the present position of knowledge on the subject, but to gain practical hints and directions as to the diagnosis and treatment of diseases of the ear and naso-pharynx will find this handsome volume a most welcome and valuable guide. Notwithstanding the number of the authors engaged in its production careful editing has resulted in a work which is harmonious throughout, and "reads" without a break. The more general subjects are taken by Macnaughton-Jones, who deals with the examination of the patient, etiology, sypmtomology, apparatus, affections of the external and internal ear, and deaf mutism. Birmingham and Joyce treat on anatomy, Stewart on the large subject of the affections of the middle ear, Milligan on suppurative complications, Tilley on the nose and naso-pharynx, while Dudley Buxton gives a short article on anæsthetics. Of Dr. Macnaughton-Jones' part in the work we need say but little; for although much of it appears to have been rewritten, he has already proved his position by the series of editions which the work has gone through. Dr. Stewart's article gives a very good account of the diseases of the middle ear; Milligan's admirable article on the complications of suppurative middle ear disease is a full and complete essay on this most important subject, which will be read with interest by his co-specialists as well as by general practitioners; Tilley's contribution on the naso-pharynx is thoroughly up to date, and shows well what is now being done by surgery for the relief of a series of morbid conconditions which are probably at the root of a great many of the diseases which come before the aural surgeon. The whole book is very well printed, is most profusely illustrated, and in every way reflects credit upon its publishers, as well as upon those by whom it has been written.

**Medical Ethics: A Guide to Professional Conduct.** By Robert Saundby, M.D.Edin. (Bristol: John Wright and Co. 1902. 3s. 6d. net.)

That there is a want of such a book as this no one can doubt, nor do we think that after reading Dr. Saundby's admirable guide anyone can reasonably complain of the manner he has been conducted over the manifold professional pitfalls the existence of which its pages lay bare. We are told in the preface that the work is founded for the most part on actual cases and decisions which have been given in the leading medical journals during the past few years, and we must confess that this statement by no means inclined us to accept its dicta without reserve, for truly the seeker after amusement can often find the object of his desire among the *ex cathedrâ* utterances of medical editors. The book, however, is better than such an introduction would suggest, and there are few emergencies in which its advice may not be followed with full confidence. Nevertheless, even in so generally good a book, we notice one or two slips. Under the heading "Change of medical attendant" it is stated that, "provided certain conditions are fulfilled, one medical practitioner may succeed another in attendance upon any person or case without affording ground for complaint," and that "the most essential condition is that the former medical attendant should be courteously informed that his services are no longer required; and it is the duty of every medical practitioner, when called upon to supersede a colleague, to take care that this act of courtesy has been performed." Now if Dr. Saundby had said that it was well or that it was desirable that he should do so we should fully agree. But it is a bad thing to make rules which must of necessity be frequently broken, and it is sufficiently obvious that when a doctor has gone several miles into the country to see a new case he cannot turn back and refuse to see the patient on finding out that the "former medical attendant" has not been "courteously informed that his services are no longer required." The courtesy with which this delicate task is performed is a matter over which doctor number two has no control, and in regard to which he should not be saddled with responsibility. In the same section Dr. Saundby no doubt accidentally commits himself to a statement so opposed to the general principles of professional reticence that we hope he will modify it when occasion arises. He says "Where a patient puts himself under the care of a new medical attendant, the latter has no right to demand from the former medical attendant details of the treatment followed by him." This, of course, is perfectly correct, but he goes on to say "but it would often be perfectly proper on the one hand to ask for, and on the other to supply, such information." We cannot agree with this. Putting on one side the seeming impertinence of such a request, there would appear to be no excuse for a former medical attendant giving to anyone, even his successor, information regarding his professional relations with his ex-patient unless by the authority of the latter. This is quite clear. Concerning consultation with homœopaths, Dr. Saundby certainly displays a greater liberality of thought than at least one of the journals from which he professes to have derived inspiration, and we will not say that he has not common sense on his side. The book is a good and useful one, and aims at a high standard of medical ethics.

